# The efficacy of penicillin in psoriasis: A retrospective chart review

**DOI:** 10.1016/j.jdin.2026.04.003

**Published:** 2026-04-13

**Authors:** Priya Kadu, Chenan A. Huang, Steven R. Feldman

**Affiliations:** a Department of Dermatology, Center for Dermatology Research, Wake Forest University School of Medicine, Winston-Salem, North Carolina; b Department of Pathology, Wake Forest University School of Medicine, Winston-Salem, North Carolina; c Department of Social Sciences & Health Policy, Wake Forest University School of Medicine, Winston-Salem, North Carolina; d Department of Dermatology, Wake Forest University School of Medicine, Winston-Salem, North Carolina

**Keywords:** penicillin, psoriasis, superantigens

*To the Editor:* Streptococcal infection triggers guttate psoriasis and may exacerbate chronic plaque psoriasis via superantigen formation. Based on the superantigen theory, penicillin may be beneficial in psoriasis. Few studies have evaluated the role of penicillin in psoriasis, and the outcomes are inconsistent.^1^, ^2^, ^3^ This retrospective study aimed to assess the efficacy of oral penicillin V potassium in psoriasis.

After obtaining institutional review board approval (IRB00127568, date approved: 3/6/2025, Advocate Health- Wake Forest University School of Medicine), the institutional database was queried to identify patients with “psoriasis” and “penicillin” between January 1st, 2000, and December 31, 2024. The inclusion criterion was patients >18 years old with psoriasis who received penicillin V potassium (penicillin VK) for psoriasis or other indications. The efficacy of penicillin was assessed retrospectively based on the patient records. The outcome was categorized into 3 classes: definitive, implied, or no improvement (Fig 1).

**Fig 1:**
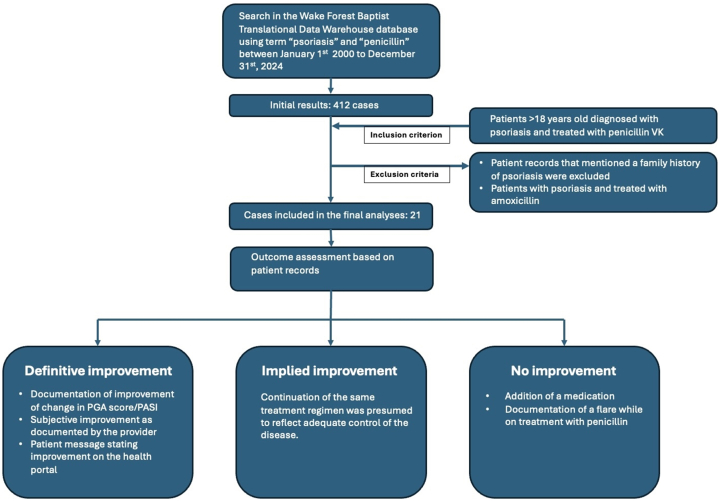
The methodology for patient selection and outcome assessment for the retrospective chart review. *PASI*, Psoriasis Area and Severity Index; *PGA*, Physician global assessment score; *VK*, V potassium.

The age range of the study cohort (10 males and 11 females) was 19 – 85 years, with a median age of 51 years (Supplementary Table I, available via Mendeley at https://data.mendeley.com/datasets/72y5jcfznb/1).

**Table I:** A Summary of clinical trials evaluating the efficacy of penicillin in psoriasis

Author	Study design	Sample size	Patient population	Intervention and outcome			Conclusion
Saxena et al^3^	Prospective open label study	36	Patients with chronic plaque psoriasis between 10 and 67 y	1.2 million IU of benzathine penicillin IM every 2 wks At 36 wk, 20 had 91% to 100%, 9 had 61% to 90% improvement, 1 had 31% to 60% improvement. (*P* < .001)			Penicillin is efficacious in chronic plaque psoriasis.
Caca-Biljanovska et al^4^	Randomized clinical trial	20	Patients with guttate psoriasis	Betamethasone dipropionate 0.05% cream and NBUVB with penicillin (*n* = 10)		Betamethasone dipropionate 0.05% cream and NBUVB without penicillin (*n* = 10)	Between the group comparison did not reach a statistical significance (*P* > .05), suggesting a lack of additional benefit with penicillin.
				Mean PASI at baseline 5.7 ± 2.1 and after 0.5 ± 0.8 6 cases had complete clearance, 2 had significant improvement and 2 had moderate improvement at 6 wk.		Mean PASI at baseline 5.9 ± 2.5 and after 1 ± 0.9 6 had complete clearance, 2 had moderate improvement, and had 2 significant improvement at 6 wk	
Dogan^5^	Randomized clinical trial	43	Patients with guttate psoriasis for >6 mo	No treatment (*n* = 15)	Oral erythromycin 250 mg 4 times daily (*n* = 14)	Oral benzathine phenoxymethylpenicillin (*n* = 14)	No clinical benefit was observed in either group after treatment. There was no statistical difference among the groups before (*P* = .322) or at 6-wk follow up (*P* = .605).
				Mean PASI scores at the baseline and at 6-wk-follow up were 11.12 and 11, respectively. (*P* = .211)	Mean PASI score at the baseline and 6-wk-follow-up were 12.6 and 11.8, respectively. (*P* = .207)	Mean PASI scores at the baseline and at 6-wk-follow up were 11.1 and 11, respectively. (*P* = .154)	

Three out of 4 cases of guttate psoriasis and 1 case of chronic plaque psoriasis tested positive, and 2 cases tested negative for streptococcal infection by rapid antigen testing. Penicillin VK was administered orally, with doses varying from 500 mg to 2000 mg daily for periods of 10 days to 20 years for various indications. 52.3% (11/21) improved after oral penicillin VK therapy. 23.8% (5/21) had definitive, 28.5% (6/21) had implied, and 42.8% (9/21) had no improvement. Nine out of 12 cases (75%) of chronic plaque psoriasis, 1 out of 4 cases (25%) of guttate psoriasis, and 1 out of 5 cases (20%) of palmoplantar pustulosis improved. No adverse effects were reported.

Despite many revolutionary medications, the treatment of psoriasis remains challenging. Penicillin remains the drug of choice for the prevention of acute rheumatic fever and its sequelae, particularly rheumatic heart disease, where an autoimmune response is triggered by superantigens and molecular mimicry. Along similar lines, penicillin may be a useful adjunct to augment long-term control of psoriasis. Many superantigens-for example, M protein, streptococcal pyrogenic exotoxin SPEA, and methicillin resistance gene (*mecA*) exacerbate psoriasis.^4^ Additionally, patients colonized with toxigenic *Staphylococcus aureus* have increased interleukin 22 levels,^5^ which facilitates epidermal hyperplasia, supporting the use of penicillin to control bacterial colonization in psoriasis.

Consistent with other prospective studies (Table I), 9 cases of chronic plaque psoriasis benefited compared to only 1 case each of guttate and palmoplantar pustulosis.

Based on the spectrum of results observed with penicillin, it may be an effective adjuvant for chronic plaque psoriasis. Patient preferences for nonimmunomodulatory, noninjection therapies, cost-effectiveness, and a good safety profile may favor its usage. Owing to the retrospective nature of the study, the results must be interpreted cautiously. The limitations include confounding with background therapies and outcome assessment (implied improvement category reflecting patient preference, medication access problems, and clinician preference rather than true benefit), a small sample size, a lack of a control group, and standardized outcome measures.

## Conflicts of interest

Dr Feldman has received research, speaking, and/or consulting support from Eli Lilly and Company, GlaxoSmithKline/Stiefel, AbbVie, Janssen, Alovtech, vTv Therapeutics, Bristol-Myers Squibb, Samsung, Pfizer, Alumis, Boehringer Ingelheim, Oruka, Amgen, Dermavant, Arcutis, Novartis, UCB, Helsinn, Sun Pharma, Almirall, Galderma, Leo Pharma, Mylan, Celgene, Ortho Dermatology, Menlo, Merck & Co, Qurient, Forte, Arena, Biocon, Accordant, Argenx, Sanofi, Regeneron, the National Biological Corporation, Caremark, Teladoc, BMS, Ono, Micreos, Eurofins, Informa, UpToDate, Verrica, and the National Psoriasis Foundation. He is the founder and part-owner of Causa Research and holds stock in Sensal Health. Drs Kadu and Huang have no conflicts of interest to declare.
